# Use of Supervised Machine Learning for GNSS Signal Spoofing Detection with Validation on Real-World Meaconing and Spoofing Data—Part I †

**DOI:** 10.3390/s20041171

**Published:** 2020-02-20

**Authors:** Silvio Semanjski, Ivana Semanjski, Wim De Wilde, Alain Muls

**Affiliations:** 1 Department of Communication, Information, Systems & Sensors, Royal Military Academy, 1000 Brussels, Belgium; alain.muls@rma.ac.be; 2 Department of Industrial Systems Engineering and Product Design, Ghent University, 9000 Ghent, Belgium; ivana.semanjski@ugent.be; 3 Industrial Systems Engineering (ISyE), Flanders Make, Ghent University, 9000 Ghent, Belgium; 4 Septentrio N.V., 3001 Leuven, Belgium; wim.dewilde@septentrio.com

**Keywords:** global navigation satellite system, spoofing, meaconing, support vector machines, principal component analysis, safety-of-life, position-navigation-timing, GPS, classification

## Abstract

The vulnerability of the Global Navigation Satellite System (GNSS) open service signals to spoofing and meaconing poses a risk to the users of safety-of-life applications. This risk consists of using manipulated GNSS data for generating a position-velocity-timing solution without the user’s system being aware, resulting in presented hazardous misleading information and signal integrity deterioration without an alarm being triggered. Among the number of proposed spoofing detection and mitigation techniques applied at different stages of the signal processing, we present a method for the cross-correlation monitoring of multiple and statistically significant GNSS observables and measurements that serve as an input for the supervised machine learning detection of potentially spoofed or meaconed GNSS signals. The results of two experiments are presented, in which laboratory-generated spoofing signals are used for training and verification within itself, while two different real-world spoofing and meaconing datasets were used for the validation of the supervised machine learning algorithms for the detection of the GNSS spoofing and meaconing.

## 1. Introduction

The open service (OS) signals of any GNSS core constellation, such as GPS, GALILEO, GLONASS, or BEIDOU, are neither encrypted nor authentication protected, and as such are vulnerable to manipulation of the Coarse/Acquisition (C/A) Pseudo Random Noise (PRN) ranging codes and the content of navigation messages. This vulnerability of the GNSS OS signals to data manipulation is irrespective of the user’s system, be it stand-alone or, as in the aviation case, an augmentation system (known as an aircraft, satellite, or ground-based augmentation system). This vulnerability of the GNSS OS signals to data manipulation, whose impact on the users is widely recognized by the industry, as shown in the literature [1,2], is prominent when used in Safety-of-Life (SoL) applications. The vulnerability of the GNSS OS signals to data manipulation in SoL applications is expressed as a risk of using manipulated data for generating Position-Velocity-Timing (PVT) solution without the system’s awareness of using hazardous misleading information. Those SoL applications include among others: aircraft GNSS-based navigation, the use of GNSS-based Automatic Dependent Surveillance Broadcast (ADS-B) for airborne traffic situation awareness, airborne spacing, separation, and self-separation applications, and GNSS-based time synchronization in traffic control systems. In this paper, we have considered two categories of data manipulation: those with data alteration, and without (genuine data preserved), which are also known as spoofing and meaconing, respectively. 

A number of different spoofing scenarios and attack modes have been thoroughly reviewed in the literature [3,4]. With most of the spoofing methods, a spoofer needs to have control over a false signal’s waveform, power levels, ranging code, and modulated data content, being consistent with the GNSS OS signals. The ideal GNSS spoofing consists of the generation and transmission of manipulated, false GNSS signals corrected for relative power levels at a given distance between the spoofer and target receiver/s, synchronized with authentic GNSS signals’ code-phase and carrier-phase, and accounted for the Doppler shift (relative motion between spoofer and target receiver) [1]. Although requiring access to the victim’s GNSS receiver (i.e., stationary GNSS-based timing receivers used in traffic control systems), a particularly subtle way to control the pseudorange measurements, and consequently performance of victim’s receiver generated PVT solution, is through the control of the receiver’s clock offset by manipulating the clock drift (time derivative of the clock offset) estimates. In most of the other spoofing scenarios, the control of the spoofing signal’s power levels (visible as an increase of C/N_0_) is required not only to overtake the authentic signal during the correlation process but also to compensate for the distance between the spoofer’s and victim’s antenna locations (in static and kinematic applications). In such cases, the pre-correlation techniques for the spoofing detection based on the monitoring of the C/N_0_ are proven weak due to the function of the Automatic Gain Control (AGC) that compensates for the fluctuation of the received signal’s power level. It was demonstrated that only 1.1 dB difference in power level between spoofed and authentic signal is sufficient to effectuate the receiver to lock onto the spoofed signal [1]. A spoofing attack can be realized either as targeting the antenna location of a static or moving receiver or an area in which target receivers are expected to operate. The meaconing consists of the recording and re-broadcasting of authentic signals with a controlled delay. 

The threat of GNSS signal spoofing proliferates with advances in the digital signal processing and hardware implementations of the Software Defined Radio (SDR) type of GNSS-spoofing transceivers [5]. This poses a significant challenge to developers of spoofing detection. Those spoofing scenarios using the open-source software-defined GPS simulator such as ‘gps-sdr-sim’ ((https://github.com/osqzss/gps-sdr-sim): Software-Defined GPS Signal Simulator that generates GPS baseband signal data streams, which can be converted to RF using SDR platforms, such as ADALM-pluto, bladeRF, HackRF, and USRP), associated with hardware implementation, such as LimeSDR ((https://limemicro.com/products/boards/limesdr): low cost, open source, apps-enabled SDR platform that can be used to support just about any type of wireless communication standard) or HackRF ((https://github.com/mossmann/hackrf): Open source hardware for (SDR)) transceivers, pose particularly serious threat to airspace users as being demonstrated by [6,7], or to road vehicle users [8]. Even the post-capture control of a UAV (Unmanned Aerial Vehicle) through the manipulation of the aircraft’s states data via a GPS spoofer has been demonstrated [9].

While various spoofing and meaconing detection and mitigation methods exist [1], those that have the potential of light implementation (especially in aviation sector) and the level of performance required in SoL applications were considered for selection by the authors of this work. Of those possible methods, the GNSS signal post-correlation method was chosen along with the premise of having the number of exploitable observables and measurements that are possible to monitor and cross-correlate by applying machine learning algorithms with purpose to detect the presence of the false signals. Contrary to the monitoring of a single (or few) estimates within the GNSS receiver, the monitoring of the cross-correlation among all available and statistically significant GNSS observables and measurements is proposed in this paper as a method of detecting the presence of the spoofing signal. 

In the existing literature, only a limited number of previous researches [10,11,12,13] on use of the Support Vector Machines (SVM) for detection of GNSS signal spoofing are known to the authors of this paper. Panice et al. [10] focused on the detection of a spoofing attack on a UAV based on the analysis of state estimation using the SVM as a tool for anomaly detection. They implement a simulation system in order to test and study the behaviour of a UAV during an attack. Their simulator generates data from a preconfigured mission that simulates false data injection from GPS. They test several scenarios, reporting the highest success rate to be 95%. Sun et al. [11] implemented a simulation scenario and computed the singular values of the wavelet transformation coefficients of the signal to form the feature vectors. Following this, they implemented three classifiers (one of which was SVM) and fused the results of all three classifiers with a K-out-of-N decision rule. The average success rate of their SVM classification was 94%, while the average success rate of the fused results was 95%. Semanjski et al. [12] applied an SVM-based approach on a simulated dataset (with matched power attack) generated with a modified Spirent GNSS signal and constellation simulator. They achieved a success rate of 96%. In the following publication [13], they implemented, for the first time, a non-simulated dataset. They integrated the synthetically generated (simulated) dataset for the SVM-based approach (for training and testing) and used data from a real-world spoofing event to validate their results. The reported success rate is 97%. 

In this paper, we depart from the existing research by applying, for the first time, the supervised machine learning-based approach that is being validated on two real-world GNSS signal manipulation events. In more detail, we present the results of the verification and validation of the supervised machine learning-based detection of GNSS signal spoofing, particularly the SVM classification. The imposed conditions for the verification were the success rate and repeatability of the classification among three synthetically generated spoofing datasets, while the successful classification using two different real-world (spoofing and meaconing) datasets were chosen for the successful outcome for the validation of the SVM-based spoofing and meaconing detection, respectively. 

The rest of the paper is organized as follows. The next section gives an overview of the datasets used in this research, together with the description of two experiments and methods used in these experiments. Section 3 gives insights into data exploratory analysis and the results of the correlation analysis, factor analysis, and the supervised machine learning-based approach. This is followed by an extensive discussion session, and the main conclusions are summarized in a separate, final section. 

## 2. Data and Method

### 2.1. Data Collection

As briefly mentioned in the Introduction section, we have used three different datasets for the development and validation of the SVM-based GNSS signal manipulation detection. Our approach includes:

(i) three synthetically generated (simulated) datasets;
(ii) a dataset that resulted from a real-world meaconing event; and
(iii) a dataset that resulted from a real-world spoofing event.

We have used code, phase, Doppler, and signal strength measurements made by Septentrio AsteRx-U, PolaRx5, and AsterRx-m2 GNSS receivers in the L1/E1/G1/B1, L2/G2/B2, L5/E5, E5a, E5b frequency bands. The data acquired are sampled at 1 Hz and are recorded in a Septentrio Binary Format (SBF), ‘Measurement Epoch’, ‘Measurement Extra’, and ‘PVT Geodetic’ measurements blocks for both laboratory generated and real-world meaconing/spoofing datasets. 

#### 2.1.1. Training Datasets

The dataset used in the supervised machine learning approach for training purposes has been completely generated in a laboratory environment. The laboratory set-up included a Spirent GNSS constellation simulator that generated signals with matching power levels, radiated over the air and received by the antenna connected to the Septentrio AsteRx-U GNSS receiver. The radiation and receiving elements of the set-up were placed inside the Wave Field Synthesis (WFS) anechoic chamber at the Fraunhofer FORTE facility [14,15]. The spoofing scenario implemented, which was previously being used by De Wilde et al. [16], was an intermediate timing attack. This timing attack has been performed by monitoring the GNSS receiver’s Pulse-Per-Second (PPS) output and affecting the receiver’s clock divergence through a programmed spoofing signal time transmission delay (emulating satellite clock drift) at a fixed rate (of 5 ns/s, 1 ns/s and 0.3 ns/s in each of three tests, respectively), with a corresponding increase in the Carrier-to-Noise Density ratio (C/N_0_) of 2 dB or more for each tracked satellite. The spoofing got enabled 180 s into the test in order to allow acquisition of both authentic and manipulated signals. In this scenario, the spoofing attack was based on manipulating the transmission time offset, inducing the pseudorange measurements error in a target (victim) receiver. The pseudorange, as determined by the target receiver, at a known physical position computed by using the spoofing signal transmission time offset, spoofer, and target receiver locations, can be mathematically described by adopting expressions and notations given by Tippenhauer et al. [4]:

(1) $$R_{ij}^{A}=\left|P_{j}-P_{i}^{A}\right|+\delta_{i}^{A}\cdot c$$

where: 

$P_{j}$ is target receiver physical location;

$P_{i}^{A}$ is spoofer physical location;

$\delta_{i}^{A}$ is spoofing signal transmission time offset; and

c is a signal propagation speed.

The timing schedule t∈{0, 780} and parameters of transmission time offset $\delta_{i}^{A}$ of the spoofing attack performed can be described by:

(2) $$\delta_{i}^{A}\left(t\right)=\{\begin{array}{cc} 0, & if\;0\;s\leq t<180\;s\\ \delta_{i}^{A}\left(t-1\right)+{\tilde{\delta }}_{i}^{A}, & if\;180\;s\leq t\leq 780\;s \end{array}$$

where: ${\tilde{\delta }}_{i}^{A}$ is programmed spoofing signal time transmission delay (5 ns/s, 1 ns/s and 0.3 ns/s, respectively) emulating satellite clock drift.

It should be noted that the transmission time offset $\delta_{i}^{A}$ in our spoofing generation procedure was defined as a constant rate of change, as opposed to the fixed value of delay given in [4]. 

A total number of 12 GPS receiving channels were used, where on the six channels the authentic (non-manipulated) GPS C/A code signal in L1 frequency band were received, while on the other six channels the manipulated (spoofed) GPS C/A code signals were received. This resulted in having both, authentic and false, GPS signals present in all three data subsets (with 948, 1493, and 4665 total epochs, respectively) for each programmed clock divergence rate value. Hereafter, this dataset is termed as the synthetically generated (simulated) training dataset.

#### 2.1.2. Validation Datasets

For the validation of the SVM-based GNSS signal manipulation detection approach, we used two different datasets of manipulated GNSS signals:

(i) the unintentional re-radiation of the authentic GNSS signal (also known as meaconing if intentional), and
(ii) the intentional radiation of the GNSS spoofing signals.

Both datasets were used as independently created validation datasets (were not a part of the model training).

The first dataset is a result of actual real-world meaconing event caused by the unintentional re-radiation of the GNSS signal over-the-air. These GNSS signals were captured by the nearby GNSS receiver. This dataset contains the following mixed authentic and spoofed signals: GPS L1-C/A, L2-P(Y), L2C, QZSS L1-C/A, L2C, GLONASS G1-C/A, G2-C/A, IRNSS L5, GALILEO E1BC, E5a, E5b, MSS L band, SBAS L1, and BeiDou B1I, B2I. In addition, the dataset contains measurements (with 459 total epochs) in the field by a Septentrio AsteRx-m2 OEM GNSS receiver. Hereafter, this dataset is termed as a meaconing validation dataset.

The second dataset is a result of an SDR-based spoofing attack dataset generated in a successful noncovered-type of spoofing attack. This dataset was produced by using the LimeSDR ((https://limemicro.com/products/boards/limesdr): low cost, open source, apps-enabled SDR platform that can be used to support just about any type of wireless communication standard) and HackRF ((https://github.com/mossmann/hackrf): Open source hardware for (SDR)) configuration with gps-sdr-sim ((https://github.com/osqzss/gps-sdr-sim): Software-Defined GPS Signal Simulator that generates GPS baseband signal data streams, which can be converted to RF using SDR platforms, such as ADALM-pluto, bladeRF, HackRF, and USRP): where live signals were combined and only became present after a simulated outage. The live signal is combined with a combiner and the outage was simulated by applying a very large attenuation to the antenna signal for a while (visible as the front-end gain value increase). During signal reacquisition, the GNSS receiver disregarded most of these spoofing signals (due to the mismatch of correlation peaks’ location), while the receiver lost its true correlation peak on GPS PRN18 and acquired a spoofed version instead. From the pseudorange measurements it is apparent that GPS satellite with GPS PRN18 got spoofed but was quickly removed by the Receiver Autonomous Integrity Monitoring (RAIM) Fault Detection and Exclusion (FDE) algorithm. The measurement of GPS L1-C/A, L1-P(Y), L2-P(Y), L2C, L5, GLONASS G1-C/A, G2-C/A, GALILEO E1BC, E6BC, E5a, E5b, E5, BEIDOU B1I, B2I, B3I, SBAS L1, L5, QZSS L1-C/A, and IRNSS L5 signals was made by a Septentrio PolaRx 5 GNSS receiver with total of 378 epochs at a 1 Hz sampling rate. In this dataset, only eight GNSS records (8 s) represent a real spoofed signal (in particular GPS PRN18) before it was removed by RAIM. Hereafter, this dataset is termed as the spoofing validation dataset.

**Figure 1:**
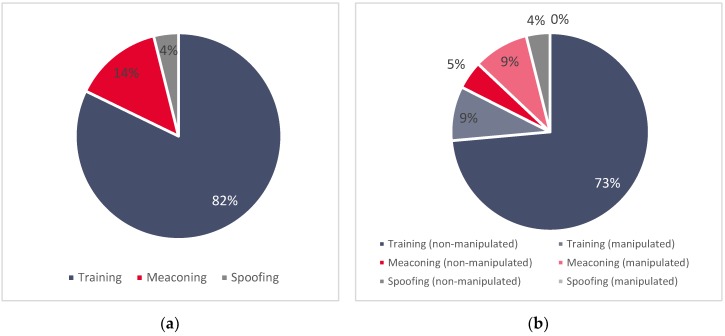
(**a**) Relative size proportions among three datasets and (**b**) the ratio of manipulated and non-manipulated GNSS signal records in each dataset.

Figure 1a shows the relative proportions among all three (simulated, meaconing, and spoofing) datasets and (b) basic characteristics of each dataset (ratio between manipulated and non-manipulated GNSS records). 

### 2.2. Methods

#### 2.2.1. Experiments

**Figure 2:**
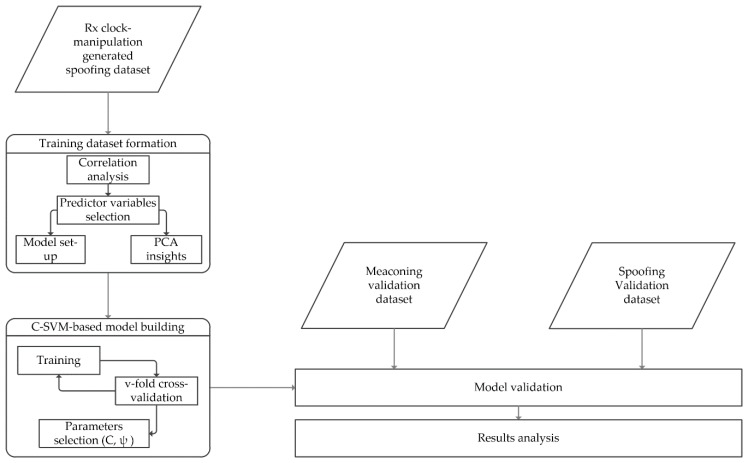
Process flow of Experiments I and II.

As indicated above, two separate experiments were performed. Experiment I includes use of intentionally spoofed (simulated) data as a training dataset for the supervised machine learning, whereas the unintentional real-world meaconing dataset was used for the testing. The same training dataset was used in the second experiment (Experiment II) as that used in Experiment I, but the validation was performed on a real-world unintentional spoofing dataset. The process flows of Experiments I and II conducted are shown in Figure 2

On this simulated training dataset, we have performed the correlation analysis. The aim of the correlation analysis was to see which variables have a statistically significant correlation with the indication if the GNSS signal was manipulated or not. Only these variables (with statistically significant correlations) were selected as predictor variables and were later used to train the supervised machine learning-based approach. In more detail, we have applied a v-fold cross-validation to obtain needed parameters for the model creation. To validate the results of the composed supervised machine learning-based model, we have used the independent validation datasets. In Experiment I, this validation dataset was the unintentional real-world meaconing dataset, while in Experiment II the validation was performed on a real-world unintentional spoofing dataset. To gain a better insight into the relations between the predictor variables and the indication if the GNSS signal was manipulated or not and to contextualize our findings, we have also applied the factor analysis. More details on the applied methods and the obtained results can be found in the following sections. 

#### 2.2.2. Correlation Analysis

As the first step, we have examined the correlation among all the observables and the indication of whether the GNSS signal is manipulated or not. For this, we have applied the well-known Pearson correlation [17,18]:

(3) $$r_{x,y}=\frac{\sum_{i=1}^{n}\left(x_{i}-\bar{x}\right)\left(y_{i}-\bar{y}\right)}{\sqrt{\sum_{i=1}^{n}{\left(x_{i}-\bar{x}\right)}^{2}}\sqrt{\sum_{i=1}^{n}{\left(y_{i}-\bar{y}\right)}^{2}}}$$

where:

- *n* is the size of the sample;

- *x_i_, y_i_* are the individual sample points indexed with i; and

- $\bar{x}$ and $\bar{y}$ are the sample means.

Only those variables that had a statistically significant correlation with the indication whether the GNSS signal was manipulated or not were considered for the following, supervised machine learning-based approach, steps. 

#### 2.2.3. Support Vector Machines Classification

To recognize the GNSS signal manipulation attempts, we opted to explore the C-Support Vector Machines (C-SVM)-based approach. The motivation for this was threefold. Firstly, C-SVM is a discriminative classifier that seems to perform plausibly in a high number of real-life applications, whether used for classification [19,20] or regression analysis [21,22]. Secondly, in the literature [12,13], there is already a provisionally supported hypothesis that C-SVM can be implemented to support the detection of GNSS signal spoofing attempts. And finally, as the detection of GNSS signal manipulation in real-life SoL applications is particularly sensitive to runtime scalability, we wanted to attain a scalable runtime in regard to the number of input samples. For this reason, we followed the findings from literature [23] that advocate using the C-SVM in applications sensitive to runtime scalability over other machine learning-based approaches, such as nu-SVM classification.

**Figure 3:**
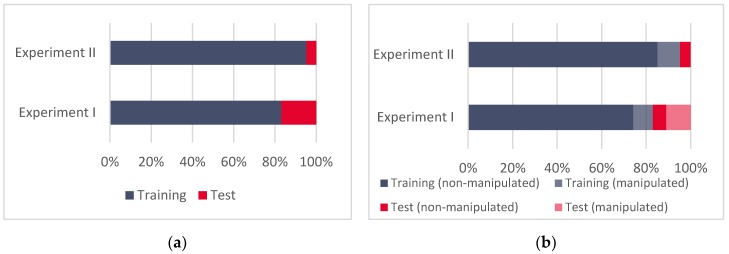
(**a**) Training and test datasets for Experiment I and Experiment II, and (**b**) the relative ratios of manipulated and non-manipulated GNSS records in datasets.

As an initial step in the supervised machine learning-based approach, we have firstly annotated the two parts of the dataset for both experiments: the training ($D_{1}$) and the test dataset ($D_{2}$). For Experiment I, this division was 83:17, and for Experiment II, 95:5 (Figure 3). 

This means that, in Experiment I, 83% of the data (the three synthetically spoofed subsets) are sorted into the training set and the remaining 17% (the meaconing validation data) into the test set. For Experiment II, the training set looked the same, but due to the different sizes of the validation dataset, the ratios were adjusted to 95% (the three synthetically spoofed subsets) for the training dataset, and the remaining 5% (the spoofing validation data) were annotated as a test dataset. Overall, Experiment I was performed over 49,860 and Experiment II over 44,651 data points. 

As C-SVM is originally designed for binary classification, to extend it to multi-class scenario (as in the GNSS signal manipulation case), we applied the one-against-all strategy and the minimization error function [20]:

(4) $$\frac{1}{2}w^{T}w+C\overset{N}{\underset{i=1}{\sum }}\xi_{i}$$

that is a subject to the constraints:

(5) $$y_{i}\left(w^{T}\;\Psi \left(x_{i}\right)+h\right)\geq 1-\xi_{i}$$

(6) $$\xi_{i}\geq 0$$

where:

i is an index that labels N training cases;

*w* is the vector of coefficients; 

*C* is the capacity constant; 

*h* is constant; 

$\xi_{i}$ represents parameters for handling non-separable data (inputs);

$y_{i}$ (y ϵ±1) represents the class labels;

*x_i_* represents the model’s independent variables; and

Ψ indicates the kernel function.

The kernel function represents a dot product of input data points mapped into the higher dimensional feature space by transformation Ψ. For our approach, we have integrated the Radial Basis Function (RBF) kernel [24,25,26] due to localized and finite responses across the entire range of the real *x*-axis:

(7) $$K\left(X_{i},X_{j}\right)=\Psi \left(X_{i}\right)\cdot \Psi \left(X_{j}\right)=\exp\left(-\psi \;{\left|X_{i}-X_{j}\right|}^{2}\right)$$

where ψ is an adjustable parameter of the kernel function.

When it comes to the C-SVM-based approach, the values of capacity constants *C* (4) and ψ (7) are particularly important to keep the training error small and to generalize well [27]. Thus, they should be chosen with care to avoid overfitting. Nonetheless, it is not possible to know upfront the best values of either of them. To overcome this challenge, we opted for the incremental grid-search across the predefined search space with a dedicated search step. For *C*, our search space for the incremental grid-search was constrained with values 1 to 10, with the search step being equal to 1. For ψ, the search space ranged from 0 to 1, with the smaller step that is equal to 10^−3^. The values obtained from the incremental search, those with the best average 10-fold cross-validation accuracy for both *C* and ψ, were chosen to be further used on the test data. 

The general idea behind the *v*-fold cross-validation is to divide the overall dataset into a number of *v* folds $A_{1},A_{2},\ldots ,A_{v}$. Hence, the *v* folds are randomly drawn disjoint sub-samples of equal sizes $(N_{1},N_{2},\ldots ,N_{v}$). The C-SVM analysis is then successively applied to the observations belonging to the *v-1* folds, which constitutes the cross-validation training sample. Hence, the *v*-fold cross-validation estimate (8) can be expressed as a proportion of cases in subsample A that are being misclassified by the classifier constructed from subsample $A–A_{v}$:

(8) $$R\left(c^{\left(v\right)}\right)=\frac{1}{N_{v}}\;\underset{(x_{n},j_{n)\in A_{v}}}{\sum }H(d^{\left(v\right)}\left(x_{n}\right)\neq j_{n})$$

where $c^{\left(v\right)}\left(x\right)$ is the classifier calculated from the subsample $A–A_{v};$

H is the indicator function for which the following is valid:

(9) $$H=\{\begin{array}{c} 1,\;\mathrm{if}\;\mathrm{the}\;\mathrm{statement}\;H\left(c^{\left(v\right)}\right)\neq j_{n})\;\mathrm{is}\;\mathrm{true}\\ 0,\;\mathrm{if}\;\mathrm{the}\;\mathrm{statement}\;H\left(c^{\left(v\right)}\right)\neq j_{n})\;\mathrm{is}\;\mathrm{false}. \end{array}$$

The results of the analyses are applied to subsample A (the fold that was not used to fit the C-SVM model) to compute the error. This error quantifies how well the observations in subsample A can be predicted by the C-SVM model. The results for the *v* replications are averaged to yield a single measure model error, i.e., the validity of the model for predicting unseen data.

Hence, we consider the test sample estimate to be the majority of cases in the test dataset that are misclassified by the classifier constructed from the learning dataset. This estimate is computed in the following way:

(10) $$E\left(c\right)=\frac{1}{N_{2}}\;\underset{(x_{n},j_{n)\in A_{2}}}{\sum }H(c\left(x_{n}\right)\neq j_{n})$$

In our approach, we applied the 10 folds cross-validation. Hence, the *v* = 10. Following this, we considered completely independent datasets, which were not used for training or for testing, where unintended meaconing and spoofing occurred in an uncontrolled environment. We applied parameters obtained from the training step in order to validate the C-SVM constructed approach.

#### 2.2.4. Principal Component Analysis

We have also applied the factor analysis, or more precisely the Principal Component Analysis (PCA), aiming to better understand the relations between the selected variables and the indication if the GNSS signal was manipulated or not. 

The PCA is a statistical procedure that uses an orthogonal transformation to convert a set of observations of possibly correlated variables into a new set of values of linearly uncorrelated variables, also called the principal components [28]. In other words, the PCA tries to explain the variance–covariance structure of a dataset using a new set of coordinate systems. This new set of coordinate systems is lesser in dimension than the number of original variables. Hence, given a set of *N* variables, a principal component model transforms variables into a new set lesser in dimension:

(11) PC<N

Yet, this equation can capture most of the variability in the original dataset. This transformation is conducted in such a way that the first principal component captures the largest possible variance, and each succeeding component, in turn, has the highest variance possible under the constraint that it is orthogonal to the preceding ones. Each coordinate, in the new transformed system, is called a principal component.

## 3. Results

### 3.1. Correlation Analysis

**Figure 4:**
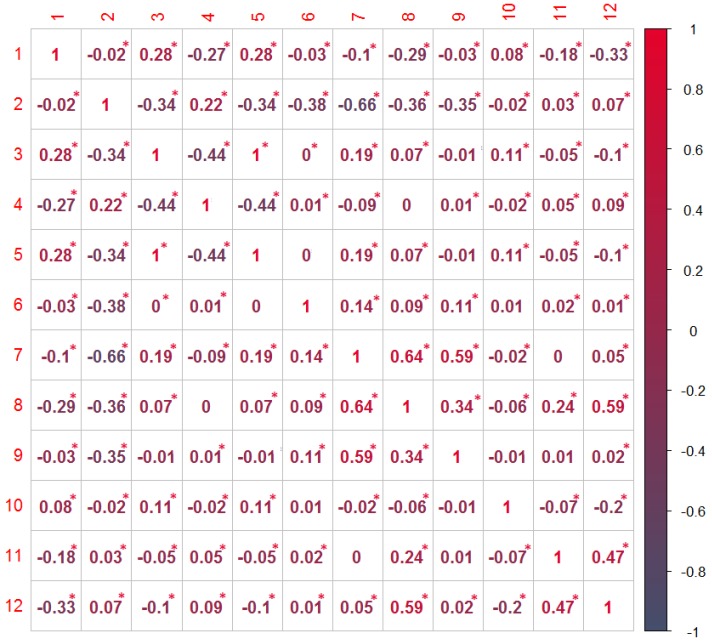
Correlation matrix (adapted from [13]).

The matrix of correlation coefficients in Figure 4 shows the partial correlations among the variables given in Table 1. Hence, the numerical value indicating the rows and the columns of the matrix corresponds to the numerical values of the “variable number” as presented in Table 1 and the value presented the matrix represents the partial correlations for corresponding variables.

The *p*-value is the probability that one would have found the current result if the correlation coefficient was in fact zero (null hypothesis). If this probability is lower than 5% (*p* < 0.05), the correlation coefficient is called statistically significant. Among the correlation values given in Figure 4, those that have statistically significant correlations (with *p* < 0.05) are marked with the red asterix. This means that we have a sufficient amount of the data and a sufficiently small *p*-value to result in a statistically relevant level of confidence in our inferences. 

### 3.2. Data Exploratory Analysis

**Figure 5:**
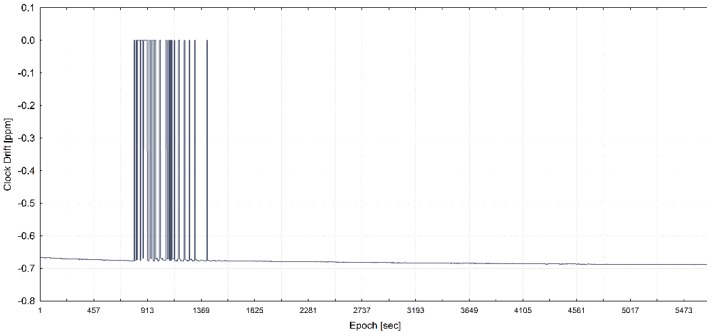
Receiver clock drift from the training dataset with a programmed 5 ns/s of time pulling (adapted from [13]).

**Figure 6:**
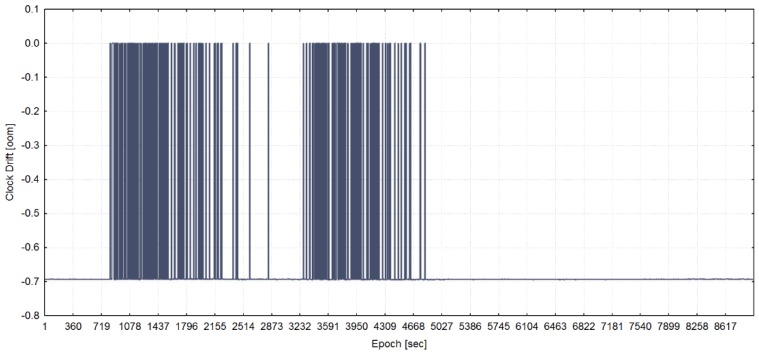
Receiver clock drift from the training dataset with a programmed 1 ns/s of time pulling (adapted from [13]).

**Figure 7:**
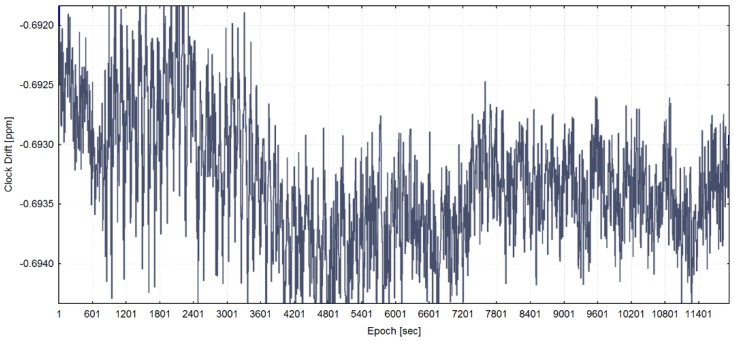
Receiver clock drift from the training dataset with a programmed 0.3 ns/s of time pulling (adapted from [13]).

The training of the model has been performed with the use of all statistically significant variables for each of three programmed receiver’s clock divergence values. In Figure 5, Figure 6, and Figure 7, the receiver clock drift is shown for each of clock divergence values (5 ns/s, 1 ns/s, and 0.3 ns/s, respectively). 

**Figure 8:**
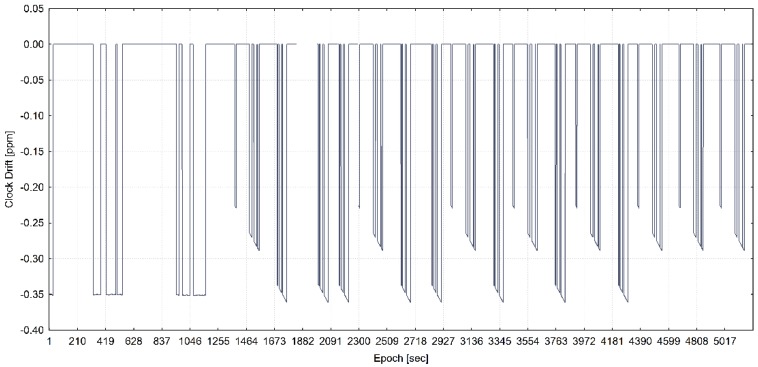
Receiver clock drift from the real-world meaconing dataset (meaconing validation dataset) (adapted from [13]).

In Figure 8, the receiver clock drift from real-world meaconing event is shown.

**Figure 9:**
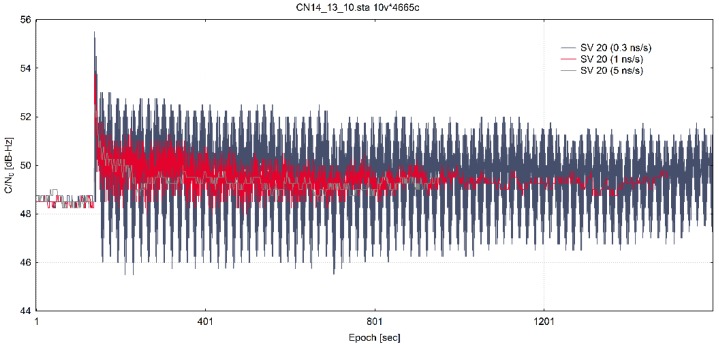
C/N_0_ of SV 20 from all three training datasets (adapted from [13]).

In Figure 9, the C/N_0_ ratio of G20 satellite is shown superimposed for all three training datasets (three clock divergence values). The start of the spoofing signal emission is visible on the graph as a jump in dB-Hz value 120 s into the test.

**Figure 10:**
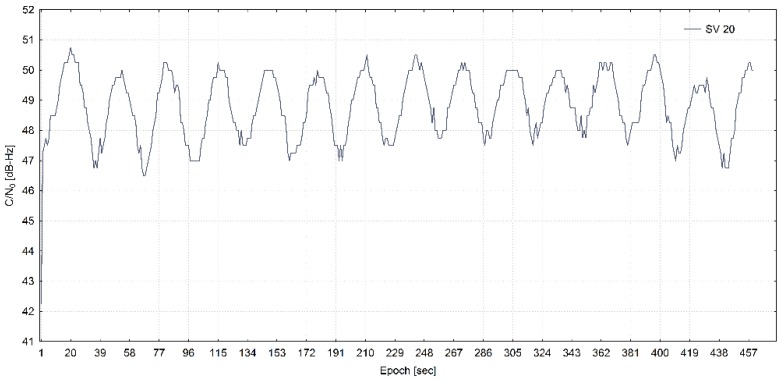
C/N_0_ of SV 20 from the real-world meaconing dataset (adapted from [13]).

The C/N_0_ density ratio for a G20 satellite (SV 20) from a real-world meaconing dataset is shown in Figure 10.

**Figure 11:**
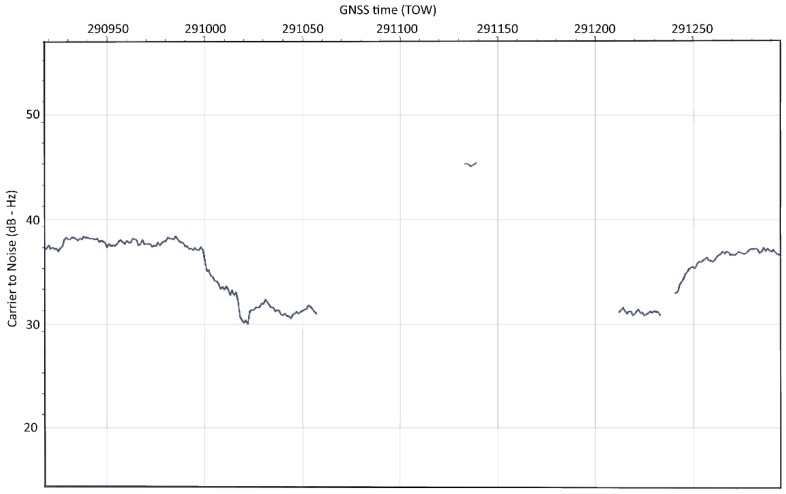
C/N_0_ of the spoofed PRN18 tracked between Time-of-Week (TOW) 291133 and 291139.

**Figure 12:**
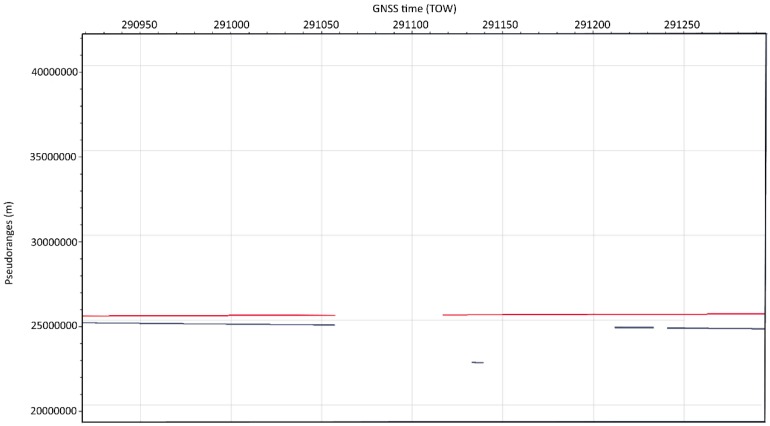
Pseudorange time plot of the spoofed GPS PRN18 (in blue) tracked between TOWs 291133 and TOWs 291139 (compared to consistent pseudorange of true GPS PRN25 in red).

In Figure 11 and Figure 12, the respective C/N_0_ and pseudorange time plots from the real-word spoofing dataset show spoofed GPS PRN18 that has been acquired for a short time after reacquisition (preceded by the forced signal outage) and tracked for the short time (six seconds) before it has been removed by the RAIM FDE algorithm.

### 3.3. Support Vector Machines

Table 2 and Table 3 give a summary of Experiment I and Experiment II results. In total, Experiment I resulted in a success rate of 98.72% with 1693 supporting vectors. Experiment II resulted in slightly less supporting vectors (1450) and a success rate of 98.77%. The 10-fold cross-validation success rated was comparable in both experiments (98.43% in Experiment I and 98.55% in Experiment II). The best values for *C* and ψ, obtained after the grid search process, were *C* = 2 and ψ = 0.75 for the Experiment I set-up and *C* = 3 and ψ = 0.8 for the Experiment II.

**Figure 13:**
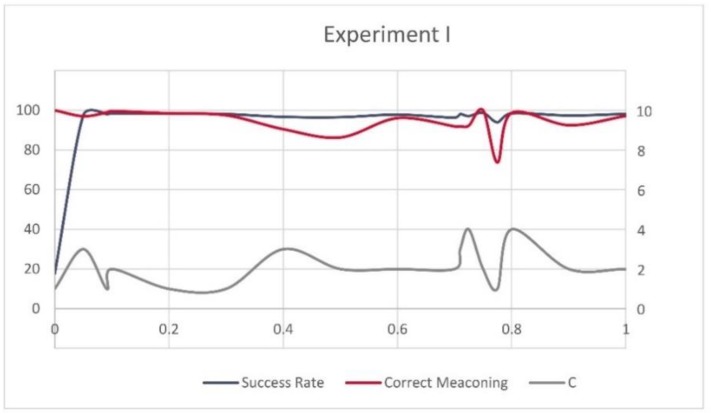
Success rate, correctly recognized meaconing records and *C* for Experiment I.

**Figure 14:**
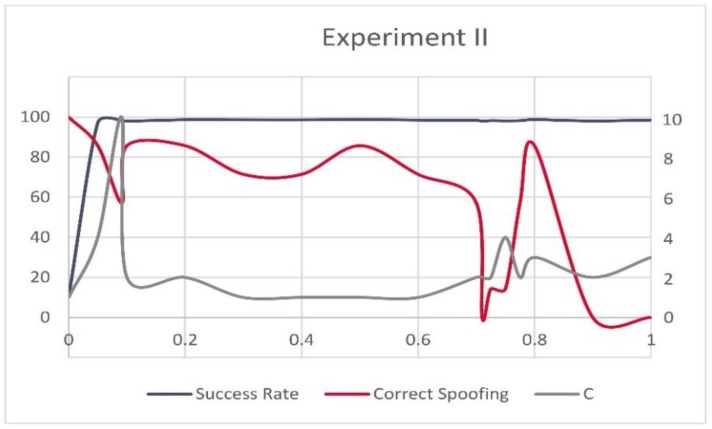
Success rate, correctly recognized spoofing records and *C* for Experiment II.

Figure 13 and Figure 14 show fluctuations between the overall success rate of the models, the success rate of the successfully identified spoofed records (left *x*-axis), and the value of *C* (right *x*-axis) in Experiment I and II in relation to the different values of ψ (*y*-axis). One can notice that the highest success rates were mainly achieved for low values of *C*. However, the high success rates of the models were not always equally translated into the high number of correctly recognized spoofed records. 

Table 4 and Table 5 show the confusion matrix for both experiments, for the validation dataset. One can notice that in the validation datasets that all the meaconing-affected data points were successfully identified, while, in the Experiment II, one spoofed data point was misclassified as an “authentic GNSS signal”.

### 3.4. Principal Component Analysis

**Figure 15:**
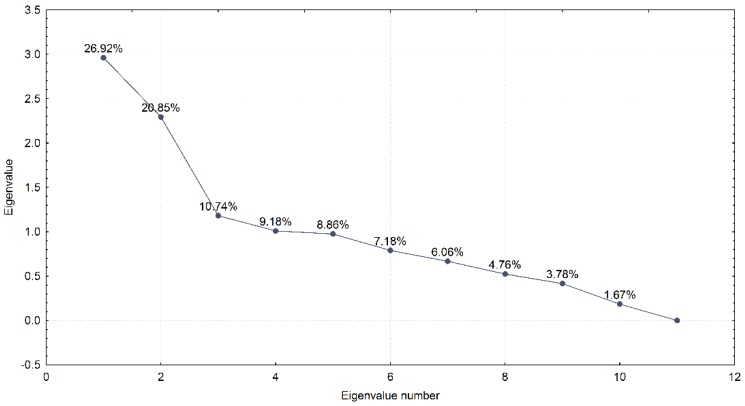
Principal components (adopted from [13]).

The results of the PCA analysis indicated 10 principal components that are shown, with the successive eigenvalues on the *x*-axis, in Figure 15. The first component accounts for almost 37% of the manipulated signal indication variations, the second for 21%, and the third for 11%. Lastly, the tenth component captures only 1.7% of the variations. Considering this, we wanted to explore which observables contributed to these factors. In the factor coordinates matrix, one can see the relation between the components (columns) and observables used to build the model (rows). This overview indicates that the first component is built mainly based on the C/N_0_, pseudorange, full carrier phase, and code variance variables (the respective eigenvalue is most correlated with these variables). Furthermore, all of the above-mentioned observables have high (and negative) correlations, with the exception of C/N_0_.

$$Factor\;coordinates=[\begin{array}{cccccccccc} -0.17 & 0.57 & -0.34 & 0.10 & -0.19 & 0.55 & -0.20 & 0.35 & 0.06 & 0.01\\ 0.77 & 0.25 & 0.23 & 0.21 & 0.03 & 0.06 & -0.03 & -0.11 & 0.44 & -0.19\\ -0.73 & 0.56 & 0.22 & -0.03 & 0.06 & -0.16 & -0.22 & -0.09 & 0.06 & -0.01\\ 0.48 & -0.44 & -0.10 & 0.03 & 0.21 & -0.19 & -0.69 & 0.12 & -0.05 & 0.01\\ -0.73 & 0.56 & 0.22 & -0.03 & 0.06 & -0.16 & -0.22 & -0.09 & 0.06 & -0.01\\ -0.24 & -0.24 & -0.42 & -0.80 & 0.06 & 0.03 & -0.02 & -0.06 & 0.24 & -0.06\\ -0.73 & -0.53 & -0.07 & 0.22 & -0.04 & -0.02 & 0.02 & 0.12 & -0.09 & -0.33\\ -0.49 & -0.60 & 0.32 & 0.11 & 0.08 & -0.07 & 0.09 & 0.36 & 0.33 & 0.15\\ -0.44 & -0.51 & -0.25 & 0.35 & -0.09 & 0.31 & -0.14 & -0.45 & 0.13 & 0.10\\ -0.08 & 0.19 & -0.26 & 0.17 & 0.91 & 0.11 & 0.15 & 0.01 & -0.01 & 0.00\\ 0.01 & -0.26 & 0.70 & -0.31 & 0.19 & 0.53 & -0.10 & -0.06 & -0.14 & -0.03 \end{array}]$$

**Figure 16:**
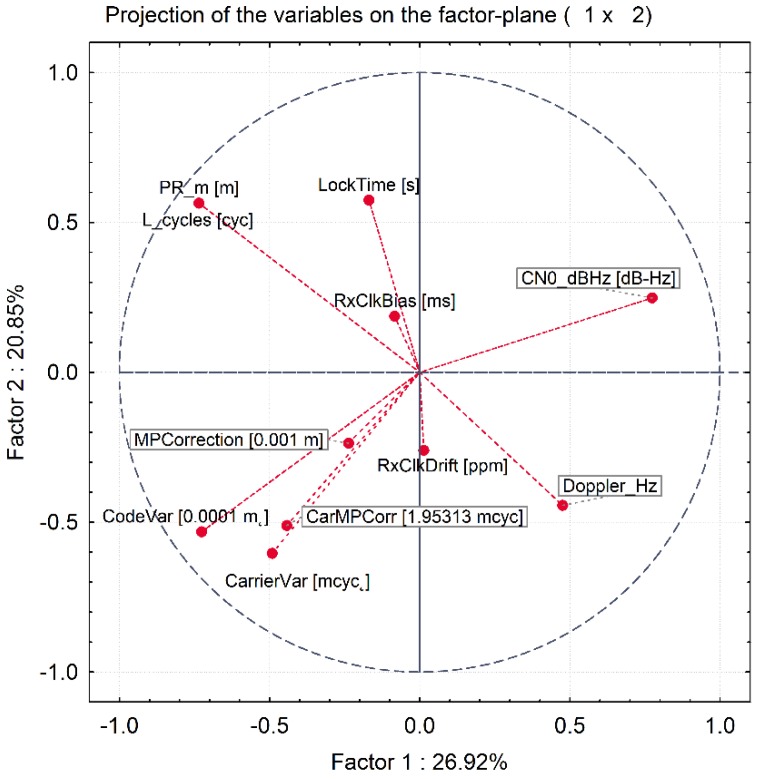
First two factors’ plot (adopted from [13]).

Figure 16 illustrates the component coordinates, in the unit circle, for the first two principal components. Considering that the analysis is based on correlations, it is evident that the largest component coordinate (variable-component correlation) that can occur is equal to 1 and that the sum of all squared principal component coordinates for a variable (i.e., squared correlations between the variable and all components) cannot exceed one. Consequently, all component coordinates must fall within the unit circle that is indicated in the graph. The closer a variable in this graph falls to the unit circle, the better its representation is by the current coordinate system. Hence, the circle provides a visual indication of how well each observable is represented by the current set of principal components. 

## 4. Discussion

As a part of the data pre-processing step, we have performed the correlation analysis across the three synthetic spoofing datasets. The aim of this process was to reduce the dimensionality of the input dataset (if possible, by excluding variables which turn out not to be correlated with the indication as to whether the signal is manipulated or not) and, on the other hand, to ensure that all the relevant variables are included in the next, C-SVM training, step. The correlation analysis indicated a statistically relevant (*p* < 0.05) correlation among the eleven variables and the indication as to whether the observed GNSS signal is manipulated or not. These eleven variables were then used as an input for the C-SVM-based training approach in both experiments (Experiment I and Experiment II). Furthermore, the principal component analysis resulted in ten principal components out of 11 variables. This indicates the high value of the selected variables as the dimensionality of the model would not significantly change if one would apply the orthogonal transformation to convert a set of observations into a set of values of linearly uncorrelated variables (components). When having a look at the principal component’s eigenvalues, it is indicative that for the first three components the percentage of the manipulated signal indication variations falls rapidly. This slope becomes less steep afterwards. Hence, one could argue that the first three components capture most of the variability and that the contribution of consecutive ones is less and less relevant.

When having a look at the C-SVM-based approach results, one can notice a relatively high number of supporting vectors involved in the separation between the authentic and manipulated GNSS observables in both experiments. This reflects the large complexity of the existing models, but can also indicate a potential overfitting. Having in mind that the approach was validated on the two independent datasets (one in each of the two experiments) being representative of the different spoofing scenarios with a high success rate achieved, the overfitting seems not to be the case. However, the high complexity still remains a challenge that needs to be tackled in future research. This is particularly relevant as it can result in a long computing time and consequently hinder the applicability of the proposed approach for the real time sensitive applications, among which any Safety-of-Life navigation application can be accounted for.

Furthermore, the validation results for the spoofing validation dataset have a slightly higher success rate (98.77%) than is the case for the meaconing validation dataset (98.72%). In Experiment I, meaconing data points were always detected, whereas in Experiment II this was not the case (one data point was not detected). Authors hypothesize that a potential reason for this could be the fact that the meaconing case is specific in two aspects: (i) the GNSS signal manipulation period is very short and partially already handled by the RAIM FDE algorithm; (ii) potentially, the meaconing is specific enough that the simulated training dataset does not provide good learning examples to successfully recognize it. In order to understand the better reasons behind these results, in Part II of this publication, we will compose new experiments and present a deeper analysis of the specifics of each dataset in order to contextualize the results. Additionally, taking into account the small number of the actually spoofed signals in this dataset (only 7 records), it is relevant to mention that the higher overall success rates were achieved during the experiment phase for values of *C* and ψ other than those used in the model, e.g., for ψ=0 and *C* = 2. However, in such cases, the number of detected spoofing signals has been zero. For this reason, in the reported results we prioritized the correct detection of the spoofing as this is critical for the SoL applications. An alternative scenario could involve prioritizing the high success rate. In the second (spoofing) validation dataset, this could be reflected in a success rate as high as 99.99%, where all except the seven actually spoofed records would be correctly classified. Hence, the reported success rate would be very high, but the actual value of such a model in practical applications would be extremely low.

Taking into the account these considerations, we find that it is highly relevant for the SoL applications to prioritize, for practical applications, the correct detection of the spoofed signal over the numerical value of the overall success rates.

Compared to the existing literature on the use of SVM-based approaches to detect GNSS signal manipulation attempts [10,11,12,13], our research presents one of a kind in the field of cyber threat analytics as it, for the first time, provides supervised machine learning-based approach insights that have been validated on the real-world spoofing and meaconing events. Next to this, we have also proposed a new approach to evaluate the success rates of the machine learning-based analytics for the SoL applications that are based on the correct detection of the manipulated signal over the numerical value of the overall success rates.

## 5. Conclusions

In this paper, we have proposed a supervised machine learning-based approach for GNSS signal manipulation detection. The proposed approach has been validated on real-world meaconing and spoofing data. The analysis of the obtained results leads to several conclusions. For one, correlation analysis seems to be a good approach for the selection of the input variables for the supervised machine learning-based approach. In our study, the PCA did not result in a major reduction in the model dimensionality when applied but has still provided beneficial insights to better understand relations among the selected input variables. Hence, it complemented the results of the correlation analysis. Next to this, the C-SVM seems to be a promising approach to detect potential GNSS signal manipulation attempts. In our two experiments, the C-SVM resulted in a quite extensive number of the supporting vectors. This indicates that the separation among manipulated and authentic signals is altogether a complex task. Furthermore, while analyzing the results of the proposed approach, we found it highly relevant for the SoL applications to prioritize, for practical applications, the correct detection of the manipulated signal over the numerical value of the overall success rates. Overall, the validation results on the meaconing and spoofing datasets seem to speak on behalf of the proposed approach as a robust one for future applications.

## Figures and Tables

**Table 1:** List of variables.

Variable number	Variable name	Unit
1	Lock time	[s]
2	C/N_0_	[0.25 dB-Hz]
3	Pseudorange	[m]
4	Carrier Doppler frequency	[0.0001 Hz]
5	Full carrier phase	[cycles]
6	Multipath correction	[0.001 m]
7	Code variance	[0.0001 m2]
8	Carrier variance	[mcycle2]
9	Carrier multipath correction	[1/512 cycle]
10	Receiver clock bias	[ms]
11	Receiver clock drift	[ppm]
12	Spoofing indication	No unit

**Table 2:** Experiment I model summary (ψ = 0.75, *C* = 2).

Experiment I	Value
Number of independents	11
SVM type	Classification type 1
Kernel type	Radial Basis Function
Number of SVs	1693 (1662 bounded)
Number of SVs (0)	845
Number of SVs (1)	848
Cross -validation accuracy	98.43%
Class accuracy (training dataset)	98.70%
Class accuracy (independent test dataset)	98.83%
Class accuracy (overall)	98.72%

**Table 3:** Experiment II model summary (ψ = 0.8, *C* = 3).

Experiment II	Value
Number of independents	11
SVM type	Classification type 1
Kernel type	Radial Basis Function
Number of SVs	1450 (1417 bounded)
Number of SVs (0)	726
Number of SVs (1)	724
Cross -validation accuracy	98.55%
Class accuracy (training dataset)	98.75%
Class accuracy (independent test dataset)	98.82%
Class accuracy (overall)	98.77%

**Table 4:** Confusion matrix for the independent meaconing validation dataset in Experiment I.

	Authentic GNSS Signal	Spoofed GNSS Signal
Authentic GNSS signal	2408	123
Spoofed GNSS signal	0	4408

**Table 5:** Confusion matrix for the independent spoofing validation dataset in Experiment II.

	Authentic GNSS Signal	Spoofed GNSS Signal
Authentic GNSS signal	1985	23
Spoofed GNSS signal	1	6
